# Stability Assessment of the Rumen Bacterial and Archaeal Communities in Dairy Cows Within a Single Lactation and Its Association With Host Phenotype

**DOI:** 10.3389/fmicb.2021.636223

**Published:** 2021-04-06

**Authors:** Zhigang Zhu, Gareth Frank Difford, Samantha Joan Noel, Jan Lassen, Peter Løvendahl, Ole Højberg

**Affiliations:** ^1^Department of Animal Science, Faculty of Science and Technology, Aarhus University, Aarhus, Denmark; ^2^Center for Quantitative Genetics and Genomics, Department of Molecular Biology and Genetics, Faculty of Science and Technology, Aarhus University, Aarhus, Denmark

**Keywords:** rumen bacteria, rumen archaea, milk phenotype, parity, dairy cows

## Abstract

Better characterization of changes in the rumen microbiota in dairy cows over the lactation period is crucial for understanding how microbial factors may potentially be interacting with host phenotypes. In the present study, we characterized the rumen bacterial and archaeal community composition of 60 lactating Holstein dairy cows (33 multiparous and 27 primiparous), sampled twice within the same lactation with a 122 days interval. Firmicutes and Bacteroidetes dominated the rumen bacterial community and showed no difference in relative abundance between samplings. Two less abundant bacterial phyla (SR1 and Proteobacteria) and an archaeal order (Methanosarcinales), on the other hand, decreased significantly from the mid-lactation to the late-lactation period. Moreover, between-sampling stability assessment of individual operational taxonomic units (OTUs), evaluated by concordance correlation coefficient (C-value) analysis, revealed the majority of the bacterial OTUs (6,187 out of 6,363) and all the 79 archaeal OTUs to be unstable over the investigated lactation period. The remaining 176 stable bacterial OTUs were mainly assigned to *Prevotella*, unclassified Prevotellaceae, and unclassified Bacteroidales. Milk phenotype-based screening analysis detected 32 bacterial OTUs, mainly assigned to unclassified Bacteroidetes and Lachnospiraceae, associated with milk fat percentage, and 6 OTUs, assigned to *Ruminococcus* and unclassified Ruminococcaceae, associated with milk protein percentage. These OTUs were only observed in the multiparous cows. None of the archaeal OTUs was observed to be associated with the investigated phenotypic parameters, including methane production. Co-occurrence analysis of the rumen bacterial and archaeal communities revealed *Fibrobacter* to be positively correlated with the archaeal genus *vadinCA11* (Pearson *r* = 0.76) and unclassified Methanomassiliicoccaceae (Pearson *r* = 0.64); *vadinCA11*, on the other hand, was negatively correlated with *Methanobrevibacter* (Pearson *r* = –0.56). In conclusion, the rumen bacterial and archaeal communities of dairy cows displayed distinct stability at different taxonomic levels. Moreover, specific members of the rumen bacterial community were observed to be associated with milk phenotype parameters, however, only in multiparous cows, indicating that dairy cow parity could be one of the driving factors for host–microbe interactions.

## Introduction

Ruminants live in a symbiotic relationship with a complex consortium of microorganisms residing in the rumen, responsible for the breakdown of feed ingredients, especially plant material rich in oligo- and polysaccharides (Flint et al., 2008). This functional feature results in the production of a wide range of microbial metabolites in the rumen, including short-chain fatty acids (SCFA), amino acids, vitamins, and methane. Some of these metabolites, in particular SCFA, represent a crucial source of nutrients to the host, contributing to the complex interaction between the rumen microbiota and host phenotypes, like milk production and composition, and feed efficiency (Vinet and Zhedanov, 2010; Jami et al., 2014; Lima et al., 2015).

Using culture-independent molecular approaches, the rumen microbiota composition has been described in a range of studies, with Firmicutes and Bacteroidetes as the dominant phyla (Pitta et al., 2014a, 2016; Jewell et al., 2015; Lima et al., 2015). Determining the driving factors for changes in the rumen microbiota composition of dairy cows has been an intense area of research, with diet as the most extensively investigated single factor (Henderson et al., 2015; Kumar et al., 2015; Nathani et al., 2015). Recent studies on large dairy cow and beef cattle cohorts have, however, identified heritable taxa of the rumen microbiota, suggesting that host genetics is also a determinant factor for rumen microbiota composition (Difford et al., 2018; John Wallace et al., 2019; Li et al., 2019). Findings like this have been a driver for hypothesizing the rumen microbiota to be composed of two key components; (i) a stable “core microbiota,” with conserved house-keeping features, crucial for functional stability of the rumen microbial community as well as the host and (ii) a “dynamic microbiota,” with an inherent plasticity allowing for adaption to swift changes in environmental stimuli, provided e.g., via the diet.

Furthermore, research characterizing the links between the variation in rumen microbiota and variation in host phenotypes, such as milk production and composition, feed efficiency, and methane emission has increased drastically. The Firmicutes to Bacteroidetes ratio has been reported to be strongly correlated with daily milk fat yield (Jami et al., 2014), and multiple bacterial taxa, including Micrococcaceae, Deltaproteobacteria, Erysipelotrichaceae, and Prevotellaceae, have been found to be correlated with milk production, and their presence to depend on cow parity and stage of lactation (Lima et al., 2015). Moreover, high feed-efficient cows have been reported to harbor a rumen microbiota with a higher relative abundances of Bacteroidetes, like *Prevotella*, and a lower relative abundance of Firmicutes (Delgado et al., 2019). Comparing the rumen microbiota composition of beef cattle varying in methane emission, revealed a higher Succinivibrionaceae abundance in low methane emitters (Wallace et al., 2015). Moreover, when testing different dosages of chloroform (an anti-methanogenic compound) to reduce methane emission, concurrent increase in the Bacteroidetes to Firmicutes ratio and a decrease in Archaea were observed (Martinez-Fernandez et al., 2016). These findings illustrate the need of cognizance of the rumen microbiome when evaluating different methane mitigation strategies on the host. Other parameters, like temporal rumen microbiota dynamics have received some research interest. For example, stage of lactation (Jewell et al., 2015; Zhu et al., 2018) and parity (Lima et al., 2015; Difford et al., 2018) have been reported to affect the rumen microbiota composition. However, temporal dynamics in rumen microbial composition e.g., caused by dietary interventions, and potential effects on associations between rumen microbiota and host phenotypes remains to be further investigated. For instance, shifts in the dairy cow rumen bacterial community over the transition period have been reported in a number of studies (Lima et al., 2015; Dieho et al., 2017; Zhu et al., 2018). In these studies, dietary effects, like switching from a high-forage prepartum to a high-concentrate postpartum diet, often dominates over host physiological status, thereby confounding dietary and host physiological changes with rumen microbial dynamics. In comparison, a relatively consistent dietary formula is typically provided to dairy cows during the lactation period, rendering a unique opportunity for obtaining insights into the temporal rumen microbial dynamics free of the dietary effects.

In the present study, we included a cohort of 60 lactating mixed-parity dairy cows, fed a standard total mixed ration, and investigated the rumen bacterial and archaeal dynamics over the lactation period by collecting rumen samples twice within a single lactation with an interval of 122 days. Rumen microbiome data were generated by 16S rRNA gene amplicon sequencing, and host phenotype data, such as milk yield, milk composition, and methane production were registered during the sampling period. We performed between-sampling stability assessment of individual bacterial and archaeal operational taxonomic units (OTUs) and investigated the associative patterns between the rumen microbial community members and selected host phenotype characteristics.

## Materials and Methods

The animal experimental procedure followed a protocol approved by The Animal Experiments Inspectorate, Danish Veterinary and Food Administration, Ministry of Environment and Food of Denmark (Approval number 2016-15-0201-00959). Sixty lactating Holstein dairy cows (27 primiparous and 33 multiparous), between 13 and 320 days in milk (DIM), were housed in the same barn at a Danish commercial farm. The farm was equipped with an automated milking robot (Lely International N.V., Maassluis, Netherlands), and the cows were rewarded with up to 3 kg of concentrate at each milking. Throughout the lactation cycle, all the cows were fed *ad libitum* with the same total mixed ration, mainly constituted of grass silage (8.6 kg/21.5 kg), corn silage (6.8 kg/21.5 kg) and crashed barley (1.3 kg), and had free access to drinking water. All individuals were sampled twice, where the first and second samplings were carried out *primo* May and *primo* September, with a time interval of 122 days. A documented rumen sampling strategy was applied by inserting a rumen flora scoop via the mouth and esophagus into the rumen (Geishauser et al., 2012). Subsequently, approximately 40 mL rumen liquid mixed with fine particles were retrieved from the rumen, poured into a 50 mL centrifuge tube, immediately put into an icebox and transferred to the lab for further handling. Aliquots (five per sample) of 1.2 mL vigorously mixed rumen liquid were transferred into1.5 mL vials, snap frozen in liquid nitrogen, and stored at –80°C until further analysis. The remaining volume of the rumen samples was stored at –20°C for SCFAs analysis.

### Milk Components Analysis and Methane Measurement

Milk yield of individual cows were recorded on the milking robot (Lely International N.V., Maassluis, Netherlands) upon each visit. On each rumen sampling day, samples of milk were withdrawn directly from the milking robot, and milk composition (protein and fat percentages) was determined using a CombiFoss (Foss, Hillerød, Denmark) at Eurofins (Vejen, Denmark). Methane production of the individual cows was measured with an on-farm methane measurement technique Gasmet DX-4000 (Gasmet Technologies, Helsinki, Finland) as described in Madsen et al. (2010) and Lassen and Løvendahl (2016). The raw data is provided in the Supplementary Material.

### Short-Chain Fatty Acids Analysis

Rumen samples, stored at –20°C, were thawed at room temperature. After vigorously mixing for several minutes, 1 mL of sub-sample was adapted to SCFAs analysis according to a previously described protocol (Canibe et al., 2007). Determination of SCFAs was performed by gas chromatography, using a Hewlett Packard gas chromatograph (model 6890; Hewlett Packard, Agilent Technologies, Nærum, Denmark) equipped with a flame ionization detector and a 30-m ZB-5 column with an internal diameter of 0.32 mm and coated with 5%-phenyl 95%-di-methylpolysiloxane with a film thickness of 0.25 μm. The raw data is provided in the Supplementary Material.

### Illumina Amplicon Sequencing and Data Analysis

All rumen samples stored at –80°C were handled in parallel and shipped on dry ice to a commercial sequencing company (GATC Biotech, Konstanz, Germany) for analysis. In order to minimize the bias in run difference, rumen samples were cross placed on 96 well plates and adapted to DNA extraction, PCR amplification and sequencing library construction. The universal bacterial 16S rRNA gene primer pairs (27 F: 5′-AGAGTTTGATCCTGGCTCAG-3′ and 534R or 518R: 5′-ATTACCGCGGCTGCTGG-3′) targeting the V1–V3 regions of 16S rRNA gene were used for studying the ruminal bacterial community. In a comparative study of different primer pairs, targeting different hypervariable regions of the bacterial 16S rRNA, this primer pair outperformed the others and provided higher species richness and diversity of the bacterial populations in the rumen (Pitta et al., 2014b). Likewise, the archaeal 16S rRNA gene primer pairs (519F: 5′-CAGCMGCCGCGGTAA-3′ and 1017R: 5′-GGCCATGCACCWCCTCTC-3′) targeting the V4–V6 regions of archaeal 16S rRNA gene were used for studying the ruminal archaeal community. One hundred twenty rumen samples from 60 cows were sequenced on the Illumina platform: for the bacterial 16S rRNA gene analysis, 36 samples were processed by Illumina HiSeq and 82 samples by Illumina MiSeq, while for the archaeal 16S rRNA gene analysis, 50 samples were processed by Illumina HiSeq and 70 samples by Illumina MiSeq. Two samples failed the bacterial 16S rRNA gene profiling. There was an inherent difference in sequencing output between the two Illumina sequencing platforms, with up to 700,000 reads produced from the Illumina HiSeq platform and up to 200,000 reads produced from the Illumina MiSeq platform. A normalization step was adopted to minimize the influence of two different sequencing platforms, and this was performed after the sequencing data analysis. The sequencing data analysis was performed with LotuS pipeline (Less OTUs scripts), developed by a research team in Belgium (Hildebrand et al., 2014). The sequence quality filtering, de-multiplexing and preparation was carried out with *sdm* options file. With the de-replication step of sequencing data, a minimum of 50 reads with exact 100% identity was applied for the bacterial sequencing data and a minimum of 10 reads with exact 100% identity was applied for the archaeal sequencing data. The total sequence length was truncated to 230 bp after barcode, adaptor, and primer removals. Sequences with homo-nucleotide over 8 were discarded. Middle quality sequences with minimum sequence length of 230 bp and minimum average quality score of 20 were used for estimating OTU abundance, while high quality sequences with minimum sequence length of 230 bp and minimum average quality score of 27 were used for OTU clustering. Filtered reads were clustered into OTUs using the UPARSE pipeline with 97% similarity (Edgar, 2013) and the OTU sequence was assigned taxonomy using RDP classifier (Wang et al., 2007). The Greengenes database (gg_13_5)^1^ was used as a reference database for taxonomy assignment. A phylogenetic tree was constructed from sequences aligned with FastTree2 (Price et al., 2009). The OTU table and tree file from the Lotus pipeline were used as input for subsequent analysis in the QIIME-1.8.0 software package. The OTU table, generated from the bacterial 16S rRNA gene profile, was normalized to 76,000 sequences for each sample and the OTU table, generated from archaeal 16S rRNA gene profile, was normalized to 70,000 sequences for each sample with the code (*single_rarefaction.py*) prior to the calculation of alpha diversity index (chao1, observed species, and PD whole tree) and beta diversity metrics (weighted UniFrac distance matrix). Consequently, six samples were excluded from the large dataset, and the remaining 114 samples from 57 cows with successful characterization of both bacterial and archaeal 16S rRNA gene profile were kept for downstream analysis. All the sequencing data were deposited in the European Nucleotide Archive (ENA) under accession number PRJEB28065.^2^

### Statistical Analysis

Based on the corresponding DIM of the cows, the 114 samples were grouped into three categories as follows: “early lactation (13–50 DIM),” “mid-lactation (50–100 DIM),” or “late-lactation (100–320 DIM).” A single-step multiple comparison procedure (Tukey’s honest significance test) was then applied to compare the relative abundance of bacterial phyla and archaeal orders across the lactation period. Significance was claimed at a *P* value < 0.05.

Lin’s concordance correlation coefficient (*C value*) is a scaled agreement index which is commonly used to assess the agreement between two measurement variables (NCSS, 2012). This index was adopted in the present study to evaluate the stability of ruminal bacterial and archaeal communities. Individual OTUs were assessed across all individual cows and the two sampling events. The calculation of concordance correlation coefficient was based on the following equation:

$$C=\frac{2\rho \sigma_{t1}\sigma_{t2}}{\sigma_{t1}^{2}+\sigma_{t2}^{2}+{\left(\mu_{t1}-\mu_{t2}\right)}^{2}}$$

where *C*, concordance correlation coefficient (between 0 and 1); ρ, Pearson correlation coefficient; σt_1_^2^, variance across all animals at the first sampling; σt_2_^2^, variance across all animals at the second sampling; μt_1_, mean value across all animals at the first sampling; and μt_2_, mean value across all animals at the second sampling. The concordance correlation coefficient (*C value*) was introduced as a parameter to evaluate the stability of individual OTUs, where *C value* >0.5 is denoted as “stable,” otherwise “unstable.”

Since replicate measures were taken per subject, a linear mixed model controlling for the random effect of subject was employed to test the significance of each bacterial and archaeal OTU fitted separately by means of the MIXED procedure in SAS (SAS 9.3, SAS Institute Inc.) as follows:

(1) $$y_{ijkl}=\mu +b_{i}\left(\dim\right)+b_{j}\left(OTU\right)+P_{k}+a_{l}+e_{ijkl}$$

where y*_*ijkl*_* is the response variable (milk production, milk fat percentage, milk protein percentage, energy corrected milk, or methane production). μ is the model intercept. The term *b*_*i*_ is the fixed regression coefficient of the linear covariate days in milk (*dim*). Term *b*_*j*_ is the fixed regression coefficient of the natural logarithm of rarified OTU counts fitted for each bacterial and archaeal OTU separately. *P*_*k*_ is the fixed effect of the *k*th parity (*k* = 1–3). Term *a*_*l*_ is the random effect parameters for each animal *a*_*l*_ ∼ND (0, σ^2^_*B*_) and e*_*ijkl*_* is the random residual error terms with distributional properties of e*_*ijkl*_* ∼ND (0, Iσ^2^_*e*_). The significance values for term *b*_*j*_ where retained and negative log transformed for *post hoc* testing using a Bonferroni correction to control for false discovery rates, the results of which can be found in Figures 4–6.

Pearson’s product moment correlations between VFA components, milk traits and bacterial taxa, were calculated by means of CORR procedure in SAS (SAS 9.3, SAS Institute Inc.). With the same program, the correlation between archaeal taxa and methane yield was analyzed. Correlations between bacterial and archaeal taxa were inferred using *SparCC* program (Friedman and Alm, 2012) in mothur 1.35.1 (Schloss et al., 2009), where the permutations parameter was set as 1000. Co-occurrence network was constructed from those taxa, with pearson correlation coefficient (Pearson *r*) >0.5 or <–0.5 and with *P* value <0.05, by Cytoscape 3.5.1 (Shannon et al., 2003). Furthermore, a Paired *t*-test was applied to compare the alpha diversity index of ruminal bacterial and archaeal communities in primiparous and multiparous cows using SigmaPlot 11.0 (SYSTAT Software, Inc).

## Results

### Composition of the Ruminal Bacterial and Archaeal Communities Over the Lactation Period

**FIGURE 1:**
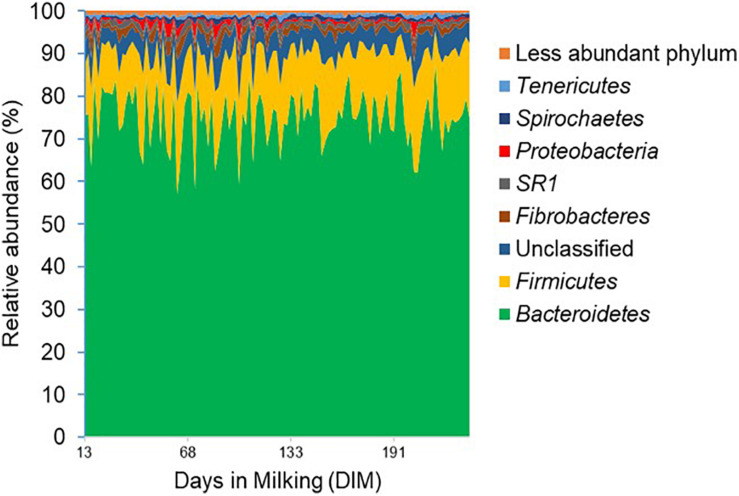
Phylum level composition of the rumen bacterial community. Bacterial 16S rRNA gene profile is shown in the area of the graph and distinct phyla are indicated by different colors. The *x*-axis represents days in milking (DIM) of cows ranging from day 13 to day 320 and the *y*-axis represents the relative abundance of bacterial taxa.

The rumen bacterial and archaeal communities were represented by 6,363 and 79 OTUs, respectively, as characterized by 16S rRNA gene amplicon sequencing. The rumen bacterial community was dominated (values in parentheses represent mean relative abundances for the two samplings) by Bacteroidetes (74.9%), Firmicutes (14.5%) and Fibrobacteres (1.5%), accounting for more than 90% of the analyzed amplicons (Figure 1) and the relative abundances of these dominant phyla did not change significantly (*P* > 0.05) over the lactation period. The ratio between Bacteroidetes and Firmicutes (B/F ratio) differed between primiparous and multiparous cows over the lactation period, with a significant decrease in B/F ratio in primiparous cows (Slope –0.0104, *P*-value < 0.05, *R*^2^ = 0.25) and a relatively constant B/F ratio in multiparous cows (Slope 0.0023, *P*-value > 0.05, *R*^2^ = 0.0048) over the lactation period (Supplementary Material).

**FIGURE 2:**
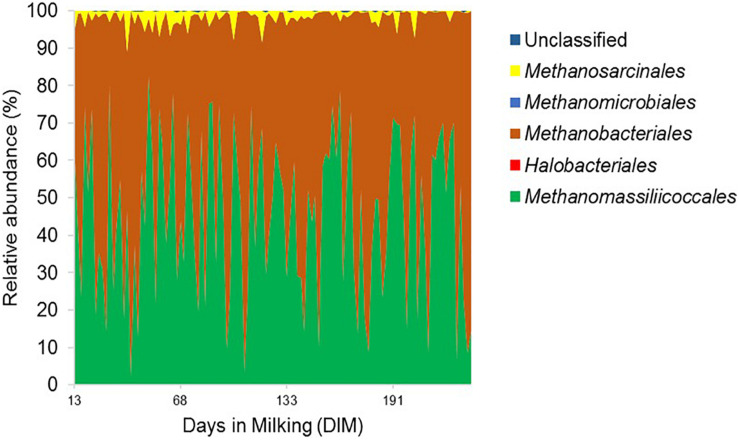
Order level composition of the rumen archaeal community. Archaeal 16S rRNA gene profile is shown in area graph and distinct orders are indicated by different colors. The *x*-axis represents days in milking (DIM) of cows ranging from day 13 to day 320 and the *y*-axis represents the relative abundance of bacterial taxa.

The relative abundance of the two least abundant phyla, SR1 (1.1%) and Proteobacteria (0.8%), on the other hand, decreased significantly (*P* < 0.05) from 1.2 and 1.3% in the mid-lactation to 0.9 and 0.6% in the late-lactation period, respectively. At the genus level, the rumen bacterial community was dominated by *Prevotella* (43.0%), *Succiniclasticum* (2.3%), *Fibrobacter* (1.5%), *SR1_genera_incertae_sedis* (1.1%) as well as a range of genera with a mean relative abundance below 1%. In comparison, the rumen archaeal community was less diverse, with Euryarchaeota being the most abundant phylum; within this phylum, five dominant orders, including Methanobacteriales (53.07%), Methanomassiliicoccales (42.73%), Methanosarcinales (1.69%), Methanomicrobiales, and Halobacteriales (both less than 1%), were identified; the least abundant two orders were only detected in a few rumen samples (Figure 2). The relative abundance of the Methanosarcinales order decreased significantly from 2.5% in the mid-lactation to 1.2% in the late-lactation period (*P* < 0.05). At the genus level, five dominant genera of the rumen archaeal community were shared by all the rumen samples, among which *Methanobrevibacter* (46.02%) was the most abundant genus, followed by unclassified Methanomassiliicoccales (27.78%), *vadinCA11* (14.95%), *Methanosphaera* (6.28%), and unclassified *Methanosarcinaceae* (1.69%).

### Diversity of the Rumen Bacterial and Archaeal Communities Over the Lactation Period

Diversity of the rumen bacterial and archaeal communities over the lactation period was assessed in multiparous and primiparous cows, separately. The diversity of the rumen bacterial and archaeal communities in primiparous cows as well as the archaeal community of multiparous cows showed no significant changes between the two samplings, however, the diversity of the rumen bacterial community in multiparous cows decreased significantly (*P* < 0.001) between samplings, as indicated by Chao1 and observed species indexes (Table 1).

**TABLE 1:** Comparison of alpha diversity index in primiparous and multiparous cows between samplings.

	Primimparous			Multiparous		
	First sampling	Second sampling	*P*-value	First sampling	Second sampling	*P*-value
***Bacteria***						
Chao1	4054.866	4076.689	0.709	4346.778	4008.709	< 0.001
Observed species	3492.875	3519.583	0.632	3747.219	3451.219	< 0.001
***Archaea***						
Chao1	36.798	39.859	0.133	40.172	40.408	0.847
Observed species	32.304	35.565	0.318	35.330	34.939	0.141

*Alpha diversity index (Chao1 and observed species) of the ruminal bacterial and archaeal communities were compared between 24 primiparous and 33 multiparous cows by paired t-test. Two samplings of the lactation period are indicated by the first sampling and second sampling. Mean values of each sampling group are shown and significant increase or decrease is defined as P < 0.05.*

### OTU-Based Stability Assessment of the Rumen Bacterial and Archaeal Communities

**FIGURE 3:**
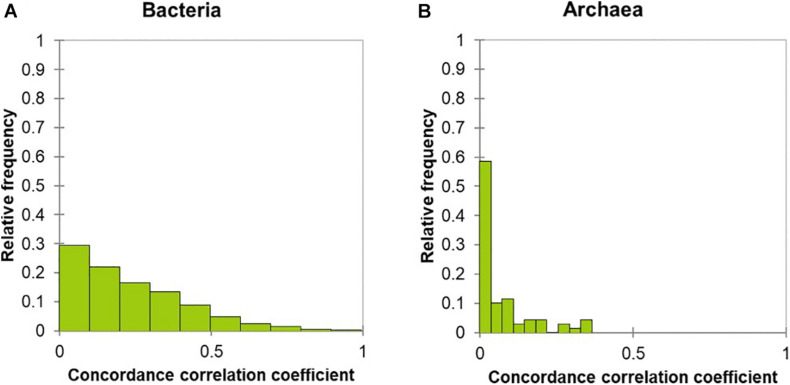
Stability assessment of the rumen bacterial and archaeal communities. The calculation of concordance correlation coefficient (C value) is described in the statistical analysis section and percentages of OTUs with similar C value are displayed. An OTU with C value greater than 0.5 and *P* value of Pearson correlation coefficient smaller than 0.05 is denoted as “stable,” as indicated by red dash line, otherwise “unstable.” Consequently, the C value distribution of 6,363 bacterial OTUs **(A)** and 79 archaeal OTUs **(B)** are shown in bar graph.

Stability assessment of individual OTUs was evaluated by analyzing the concordance correlation coefficient (C-value), ranging from 0 to 1, with a C-value of 1 indicating complete stability; a C-value of 0.5 was chosen as the threshold between “stable” and “unstable.” The relative frequency of the 6363 bacterial OTUs with a C-value above 0.5 was 2.7 % (Figure 3A and Supplementary Figure 1). In other words, this assessment resulted in 176 bacterial OTUs (~2.7% of a total number of 6,363 bacterial OTUs) being stable over the course of the two samplings, whereas the remaining 6,187 bacterial OTUs were unstable. Of those 176 OTUs, 116 OTUs were detected in more than a half of the samples, with an average number of reads between 1 and 1,127 across all the samples, while the remaining 60 OTUs were detected in less than a half of the samples. The 176 stable OTUs were taxonomically assigned to *Prevotella* (36 OTUs), unclassified Prevotellaceae (27 OTUs), unclassified Bacteroidales (25 OTUs), unclassified Bacteroidetes (16 OTUs), unclassified Lachnospiraceae (15 OTUs), unclassified Clostridiales (9 OTUs), unclassified Alphaproteobacteria (4 OTUs), *Ruminococcus* (2 OTUs), and others (42 OTUs). In contrast, none of the 79 archaeal OTUs was stable over the course of the two samplings; all had C-values below 0.5 (Figure 3B).

### Milk Phenotype-Based Screening Analysis of Bacterial OTUs

**FIGURE 4:**
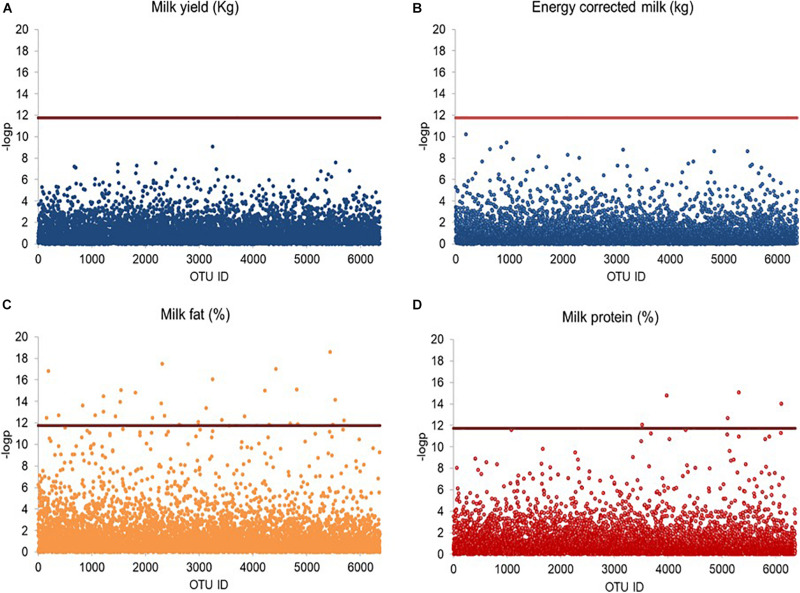
Scatter plot illustrating OTUs significantly associated with milk traits. Rarified sequence number of individual OTUs was used as input for the linear mixed model-based significance detection. Negative log base 10 transformed *P* value of individual OTUs (*y*-axis) was plotted against their corresponding OTU identities (*x*-axis). Bonferroni corrected false discovery threshold (0.05/6,363) is used as baseline for significance control as indicated by red line and individual OTUs are represented by open blue circle. The OTUs above the red baseline are considered as OTUs with significant effect on milk traits. **(A)** Milk production, **(B)** energy corrected milk, **(C)** milk fat percentage, and **(D)** milk protein percentage are shown in Manhattan plots.

Linear mixed model-based screening analysis indicated that none of the 6,363 bacterial OTUs was significantly associated with milk production and energy corrected milk yield (Figures 4A,B); however, 32 OTUs were significantly associated with milk fat percentage (Figure 4C), and 6 OTUs were significantly associated with milk protein percentage (Figure 4D), all with a relative abundance below 0.5%. The 32 milk fat percentage-associated OTUs were taxonomically assigned to Lachnospiraceae (11 OTUs), Ruminococcaceae (2 OTUs), Porphyromonadaceae (2 OTUs), Prevotellaceae (1 OTU), unclassified Clostridiales (3 OTUs) and unclassified Bacteroidales (1 OTU), while the remaining OTUs were assigned to unclassified Bacteroidetes (10 OTUs) and unclassified Bacteria (2 OTUs). At genus level, 3 OTUs were further assigned to *Butyrivibrio* (1 OTUs) and *Porphyromonas* (2 OTUs). The six-milk protein percentage-associated OTUs were taxonomically assigned to *Ruminococcus* (1 OTU), unclassified Ruminococcaceae (1 OTU), unclassified Lachnospiraceae (1 OTU) and unclassified Bacteria (3 OTUs).

**FIGURE 5:**
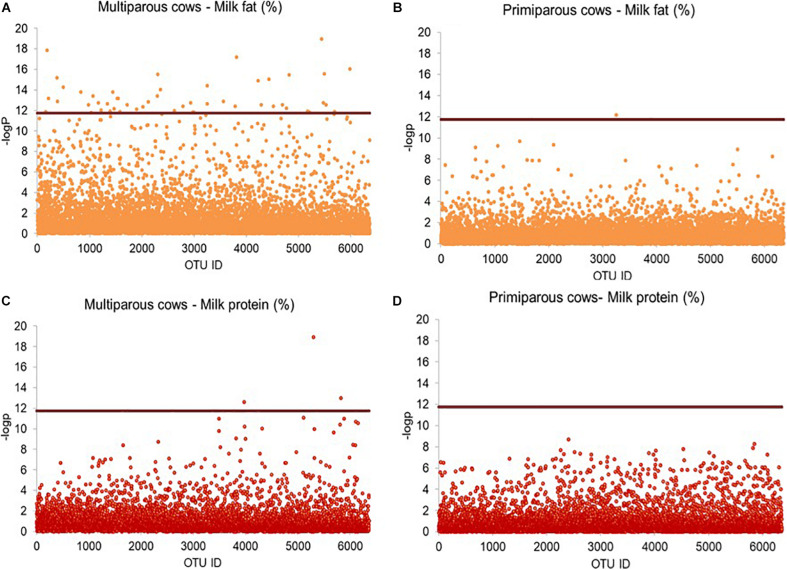
Comparison between multiparous and primiparous cows in OTUs significantly associated with milk traits. Rarified sequence number of individual OTUs was used as input for the linear mixed model-based significance detection. Negative log base 10 transformed *P* value of individual OTUs (*y*-axis) was plotted against their corresponding OTU identities (*x*-axis). Bonferroni correlation (0.05/6,363) is used as baseline for significance control as indicated by red line and individual OTUs are represented by open blue circle. The OTUs above the red baseline are considered as OTUs with significant effect on milk traits. Milk fat percentage of **(A)** multiparous cows and **(B)** primiparous cows, milk protein percentage of **(C)** multiparous cows and **(D)** primiparous cows are shown in scatter plots.

Given the differences in milk quantity and quality between multiparous and primiparous cows, we separated their OTU profiles from each other, rendering a slightly higher number of bacterial OTUs in multiparous cows (6,363 OTUs) than in primiparous cows (6,355 OTUs). The need to perform the separated screening analysis was emphasized by the finding of 51 out of 6,363 bacterial OTUs, significantly associated with milk fat percentage (Figures 5A,B), and 3 out of 6,363 bacterial OTUs, significantly associated with milk protein percentage, in multiparous cows, whereas none of the 6,355 identified bacterial OTUs in primiparous cows showed any significant associations with milk phenotypes (Figures 5C,D). The 51-milk fat percentage-associated OTUs were taxonomically assigned to Lachnospiraceae (16 OTUs), Porphyromonadaceae (5 OTUs), Ruminococcaceae (2 OTUs), Prevotellaceae (2 OTUs), while the remaining 26 OTUs were mainly assigned to unclassified Bacteroidetes (14 OTUs) and others (12 OTUs). Among those OTUs, 7 OTUs were further classified as *Porphyromonas* (4 OTUs), *Butyrivibrio* (2 OTUs), and *Prevotella* (1 OUT). The three-milk protein percentage-associated OTUs were assigned to unclassified Ruminococcaceae, unclassified Alphaproteobacteria and unclassified Bacteria.

### Methane Production-Based Screening Analysis of Archaeal OTUs

**FIGURE 6:**
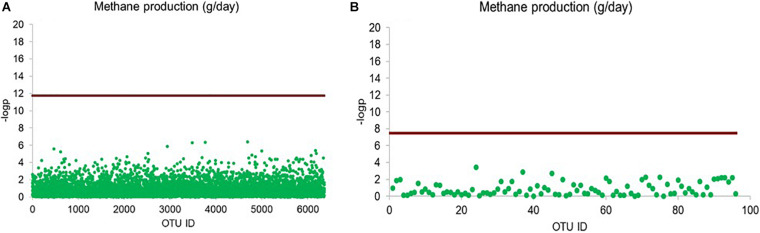
Scatter plot illustrating bacterial and archaeal OTUs significantly affecting methane production (g/day). Rarified sequence number of individual OTUs was used as input for the linear mixed model-based significance detection. Negative log base 10 transformed *P* value of individual OTUs are shown in *y*-axis and their corresponding identities are shown in *x*-axis. Bonferroni correction is used as baseline for significance control, bacterial OTU (0.5/6363) **(A)** and archaeal OTU (0.5/79) **(B)**, as indicated by red line and individual OTUs are represented by green dot.

Linear mixed model-based screening analysis did not reveal any significant associations between the 79 archaeal OTUs and methane production (Figure 6).

### Correlation Analysis Between SCFAs, Milk Traits and Genus Abundance

**FIGURE 7:**
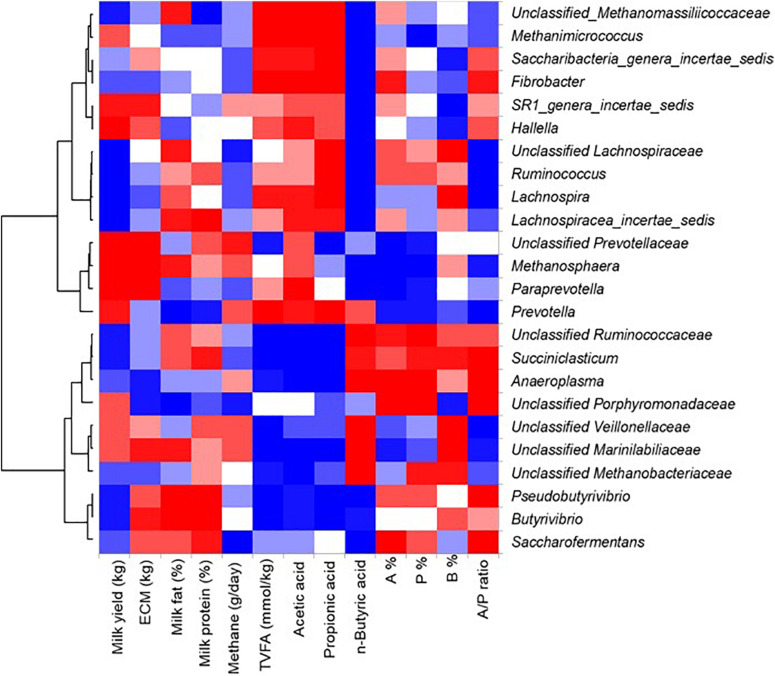
Correlation between SCFAs, milk traits and genus abundance. Pearson correlation analysis between dominant bacterial genera (with relative abundance greater than 0.1%) and concentrations (mmol/kg wet sample) and proportions (%) of SCFAs, milk components (milk yield, energy-corrected milk yield (ECM), milk fat percentage, and milk fat percentage), and methane production (g/day) across the two samplings was performed and the results are shown in Heat map. Only those bacterial taxa showing significant correlations (*P* < 0.05), positive (red) and negative (blue), with either of the listed items on the left side are shown.

Correlation analysis was performed to evaluate the relationship between rumen microbial taxa, rumen SCFA profiles, and milk traits, and only those microbial taxa with a relative abundance of more/higher than 0.1% were included in the analysis. Furthermore, the taxa not showing any significant correlation to either of the tested parameters were excluded from the results (Figure 7). Thus, Saccharibacteria showed the most positive correlation (Pearson *r* = 0.38), whereas unclassified Ruminococcaceae showed the most negative correction to total SCFA concentration (Pearson *r* = –0.33), with a similar correlation pattern for the two genera to acetate concentration (Pearson *r* = 0.39 and -0.40, respectively). In addition, Saccharibacteria showed the strongest correlation to both acetate (Pearson *r* = 0.39) and propionate concentration (Pearson *r* = 0.43), and negative correlation to butyrate concentration (Pearson *r* = –0.66). Unclassified Ruminococcaceae and unclassified Veillonellaceae showed a positive correlation to butyrate concentration (Pearson *r* = 0.37 and 0.31, respectively). The remaining microbial taxa (e.g., unclassified Marinilabiliaceae, unclassified Porphyromonadaceae, *Methanosphaera* and *Lachnospira*) showed moderate correlations with individual SCFA proportions (Pearson r between –0.25 and 0.26). Similarly, several taxa (e.g., *Hallella*, *Ruminococcus*, *Saccharibacteria, Saccharofermentans* and unclassified Porphyromonadaceae) showed positive correlations to A/P ratio (Pearson *r* ranging from 0.19 to 0.28). Among all taxa, unclassified Veillonellaceae showed the most negative correlation to the A:P ratio (Pearson *r* = –0.43).

Unclassified Prevotellaceae and *Hallella* (both Bacteroidetes) were positively correlated with milk yield (Pearson *r* = 0.21 and 0.33, respectively) and energy-corrected milk yield (Pearson *r* = 0.19–0.21, respectively), whereas *Lachnospiraceae_incertae_sedis* and unclassified Ruminococcaceae (both Firmicutes) were negatively correlated with milk yield (Pearson *r* = –0.22 and –0.25, respectively). *Butyrivibrio* showed the most positive correlation to milk fat percentage (Pearson *r* = 0.44), whereas *Prevotella* showed the most negative correlation to milk fat percentage (Pearson *r* = –0.22). Three Firmicutes genera (*Butyrivibrio*, *Lachnospiraceae_incertae_sedis*, and *Pseudobutyrivibrio*) showed positive correlations with milk protein percentage (Pearson *r* ranging from 0.23 to 0.30).

### Co-occurrence Analysis of Bacterial and Archaeal Taxa

**FIGURE 8:**
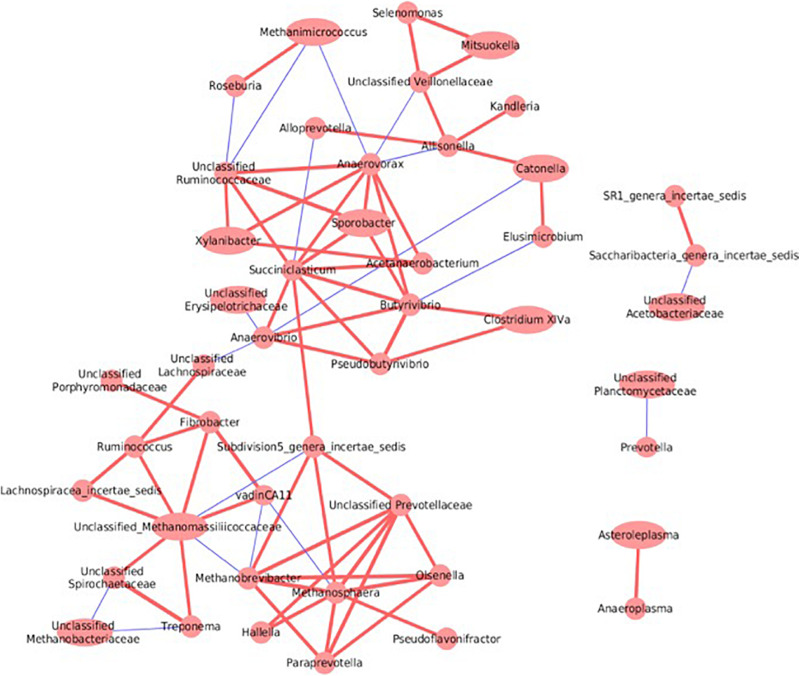
Co-occurrence network analysis between rumen bacterial taxa and archaeal taxa. Only correlations with coefficient >0.5 or <–0.5 and with *P* value <0.05 were displayed. The width of circle corresponds to the relative abundance of individual taxa. Different line colors are used to indicate a positive (red) or negative correlation (blue) between two taxa and the strength of correlation is indicated by the thickness of the lines.

Complex interactions both between and within the rumen bacterial and archaeal communities were revealed by co-occurrence analysis (Figure 8). For example, *Fibrobacter* was positively correlated with *vadinCA11* (Pearson r = 0.76); *Ruminococcus*, along with *Lachnospiraceae_incertae_sedis* and *Fibrobacter*, were positively correlated with unclassified Methanomassiliicoccaceae (Pearson *r* = 0.53, 0.63 and 0.64, respectively). Within the rumen archaeal community, both *Methanosphaera* and *Methanobrevibacter* were negatively correlated with *vadinCA11* (Pearson *r* = –0.53 and –0.56); *Methanobrevibacter* was also negatively correlated with unclassified Methanomassiliicoccaceae (Pearson *r* = –0.54). In addition, betweenness centrality score of the co-occurrence network was used to identify keystone species; a high score of a taxon reflects the importance of control over the interactions of other taxons in the rumen microbial network. As a result, *Saccharibacteria* and *Succiniclastium* of the rumen bacterial community had the highest centrality score (1.0 and 0.50, respectively); similarly, unclassified Methanomassiliicoccaceae and *Methanosphaera* of the rumen archaeal community had the highest centrality score (0.27 and 0.12, respectively).

## Discussion

### Dynamic Shift of the Rumen Bacterial and Archaeal Communities Over the Lactation Period

At the phylum level, we observed Bacteroidetes and Firmicutes to be dominating the rumen bacterial community and stable in their relative abundance across individual cows and irrespective of parity, DIM, and sampling time, in accordance with previous studies (Pitta et al., 2014a; Jewell et al., 2015; Lima et al., 2015). Likewise, Methanobacteriales and Methanomassiliicoccales were dominant orders of the rumen archaeal community, as also previously reported (Seedorf et al., 2015; Zhu et al., 2017). However, the significant decrease in relative abundances of two bacterial phyla (Proteobacteria and SR1) and one archaeal order (Methanosarcinales) between the two samplings, suggest that the rumen bacterial and archaeal communities (as evaluated on the phylum level) cannot simply be assumed to be completely stable over the lactation period.

The repeated sampling within a single lactation period allowed us to assess the stability of individual OTUs. The choice of the C-value threshold of 0.5 is arbitrary and motivated by being precisely in between perfect concordance (C-value = 1) and complete lack of concordance at (C-value = 0). Resultantly, our observation of unstable bacterial OTUs, along with decreases in the relative abundance of the two phyla, are in agreement with previous findings that the rumen bacterial communities of dairy cows, under the same management system, differed in their composition (Jewell et al., 2015; Bainbridge et al., 2016). Lactation stage has been considered as an important factor responsible for shifts in the rumen bacterial communities of lactating dairy cows (Jewell et al., 2015; Bainbridge et al., 2016) and transition cows (Pitta et al., 2014a; Lima et al., 2015; Dieho et al., 2017; Zhu et al., 2018), and likewise for parity (Pitta et al., 2014a; Jewell et al., 2015; Lima et al., 2015). Accordingly, the fact that the dairy cows involved in the present study varied in lactation stage and parity may account for the observed differences in the rumen bacterial community composition.

Likewise, individual archaeal OTUs-based stability assessment, along with changes in the relative abundance of the order Methanosarcinales, demonstrated an unstable methanogenic archaeal community over the lactation period. The rumen methanogen community is in general resilient to changes in dietary composition and all the animals were in our case maintained on the same diet over time. Diets with different forage-to-concentrate ratios (80:20, 60:40, 40:60, and 20:80) have been observed to have no significant effects on the rumen archaeal community structure at genus level, but significant differences were observed at OTU level between dietary treatments (60:40 versus 40:60) (Zhang et al., 2017). This information suggests that individual OTU-based analysis can provide different insights into the rumen methanogenic archaeal community. It has been reported that the rumen methanogen community was more influenced by DIM than breed differences (Cersosimo et al., 2016). More specifically, the mean relative abundance of *Methanosphaera* was highest around mid-lactation and lowest around early- and late-lactation, further iterating that the difference in DIM (lactation stage) of the host plays a critical role in the changes of the rumen methanogen community (Cersosimo et al., 2016). In addition, age-related compositional and functional investigations of the rumen methanogenic community revealed that methylotrophic methanogenesis was more active in the young animal rumen, whereas hydrogenotrophic methanogenesis was more active in the mature animal rumen (Friedman et al., 2017). Potential age- or parity-related changes in the rumen methanogen community should thus be taken into consideration. Again, we presented here an unstable rumen methanogen community of dairy cows over the lactation period.

### Stable Feature of the Rumen Bacterial Community

Despite the shifts of the rumen bacterial community composition at the phylum and OTU level, we identified 176 stable bacterial OTUs, among which 36 OTUs were taxonomically assigned to *Prevotella*, representing the most prevalent genus in the rumen. Functionally, members of *Prevotella*, like *Prevotella ruminicola* (former *Bacteroides ruminicola*), have demonstrated abilities for xylan, starch, pectin, and peptide degradation (Dehority, 1966, 1969; Cotta, 1988; Kim et al., 2017). A recent multi-omics study revealed positive correlations between mainly *Prevotell*a species and ruminal microbial metabolites related to amino acid and carbohydrate metabolism (Xue et al., 2020). The functional flexibility may allow them to adjust to the challenging rumen conditions. Additionally, it has been reported that the rumen microbiota can display substantial redundancy (overlap of function among multiple species/taxa) and resilience (resistant to perturbation) (Weimer, 2015). These features may be related to the genetic diversity of the *Prevotella* group, potentially rendering members of the genus functionally flexible (Avgustin et al., 1994). Further efforts are still required to isolate and characterize members of this taxon. Other stable OTUs were assigned to unclassified Prevotellaceae (27 OTUs), unclassified Bacteroidales (25 OTUs), and unclassified Lachnospiraceae (15 OTUs); these bacterial taxa have been suggested as part of the “core bacterial microbiome” in ruminants (Henderson et al., 2015). Multiple OTUs belonging to the Prevotellaceae, Lachnospiraceae, and Bacteroidales, were correlated with gross feed efficiency of dairy cows (Jewell et al., 2015), suggesting that the stable OTUs (bacterial species) we observed may possess important and conserved ecological and functional implications to the ruminant host.

### Association Between Microbial Taxa, SCFAs and Milk Components

Our linear mixed model-based screening analysis revealed that the OTUs (32 in total), significantly associated with milk fat percentage were taxonomically assigned to Bacteroidetes [e.g., unclassified Bacteroidetes (10 OTUs)], as well as Firmicutes [e.g., unclassified Lachnospiraceae (9 OTUs) and *Butyrivibrio* (2 OTUs)]. This observation is in alignment with the results from correlation analysis between bacterial taxa abundance and milk fat percentage, such as *Lachnospiraceae_incertae_sedis* and milk fat percentage (Pearson *r* = 0.20), *Butyrivibrio* and milk fat percentage (Pearson *r* = 0.44), and *Pseudobutyrivibrio* and milk fat percentage (Pearson *r* = 0.28). These observations may be attributable to their abilities to produce specific SCFAs, as indicated by the positive correlations between *Lachnospiraceae_incertae_sedis* abundance and concentrations of acetate (Pearson *r* = 0.21) and propionate (Pearson *r* = 0.20). Acetate can be incorporated into a wide range of microbial fatty acids, and propionate and butyrate can serve as substrate for lipogenesis to some extent (Emmanuel et al., 1974). However, despite the dominance of *Prevotella* in the rumen microbiota and its positive correlations with total and individual SCFA concentrations, this genus showed the most negative correlation with milk fat percentage (Pearson *r* = –0.22). This observation is comparable to a previous finding that the abundance of *Prevotella*, was negatively correlated with milk-fat yield (Pearson *r* = –0.69) (Jami et al., 2014). On the contrary, other studies reported a positive correlation between *Prevotella* and milk fat percentage (Chiquette et al., 2008; Indugu et al., 2017). One explanation of this discrepancy could be due to the dilution effect of milk. We observed a positive correlation between *Prevotella* and milk yield, as also reported by another study (Indugu et al., 2017). A negative correlation between milk yield and milk fat percentage was found when we examined the relationship between these two parameters at host level (data not shown). This information suggests that an increase in the total quantity of milk may lead to a decrease in milk fat percentage. Another explanation of this discrepancy could be related to the genetic diversity of *Prevotella*, with the inclusion of different functional species occupying different metabolic niches (Bekele et al., 2010). Varied responses of individual genus members to dietary components may contribute to the negative correlation between *Prevotella* and milk fat percentage. The genus *Pseudobutyrivibrio* harbors butyrate-producing species (e.g. *Pseudobutyrivibrio xylanivorans* (Kopečný et al., 2003), and within the genus *Butyrivibrio*, *Butyrivibrio fibrisolvens* even plays a major role in regulation of rumen lipid metabolism (Kepler et al., 1966; Maia et al., 2010).

Linear mixed model-based analysis revealed that 6 OTUs, related to *Ruminococcus*, unclassified Ruminococcaceae, unclassified Lachnospiraceae and unclassified Bacteria, were significantly associated with milk protein percentage. *Ruminococcus* and unclassified Lachnospiraceae are not only abundant members of the rumen microbial community, but also play a very important role in fermenting diverse plant polysaccharides. For instance, two important cellulolytic members of the *Ruminococcus* genus, *Ruminococcus flavefaciens* and *Ruminococcus albus*, can degrade complex plant polysaccharides into oligosaccharides (Krause et al., 2003); this fundamental step in the rumen fermentation scenario may affect the growth and activity of other members of the rumen microbial ecosystem, thereby regulating the ruminal microbial biomass.

Interestingly, only two OTUs were shared between the milk phenotype-associated OTUs and the stable OTUs (Supplementary Figure 1), suggesting these OTUs (bacterial species) may fill different functional roles in the rumen microbial ecosystem. Most of the stable OTUs were taxonomically assigned to *Prevotella* and unclassified Prevotellaceae, and a multitude of correlations were detected between *Prevotella*, along with *Lachnospira, Succiniclasticum*, and *Saccharofermentans*, SCFAs, and milk traits. It is possible that these stable OTUs are fundamental for the rumen anaerobic food chain, while milk-phenotype associated OTUs may be functionally active in the rumen. A stable profile of individual SCFA proportions was observed across individual animals, parity and lactation stage (Supplementary Figure 2), indicating that overall, the rumen microbial activity was not affected by shifts in the rumen microbial community structure over the lactation period. These observations may be closely related to the capacities of the stable *Prevotella* OTUs (species) to degrade a wide variety of feed ingredients into SCFAs (Dehority, 1966; Cotta, 1988). On the other hand, pectin degradation by *Lachnospira* species (Rode et al., 1981) and glucose degradation by *Saccharofermentans* species (Chen et al., 2010) were also enhanced. Perhaps *Prevotella* species may act as primary degraders of feed ingredients, providing substrates for other functionally active bacteria species which may further contribute to milk phenotype. However, due to lack of cultured isolates, the understanding of the metabolic functions and networks of these, as well as a range of unclassified groups is still in its infancy. Therefore, taking advantage of type species or strains obtained from existing culture collections or novel culturomics approaches to gain mechanistic insights into the host-microbe interactions through more advanced computational approaches such as genome-scale metabolic modeling is highly encouraged (Perez-Garcia et al., 2016).

A large number of the milk phenotype-associated OTUs, identified from the entire dataset, were only found in the multiparous cows and not the primiparous cows, suggesting parity to be a driver of the interactions between the rumen microbiome and milk phenotype. Indeed, previous studies have reported significant differences in milk production (Wathes et al., 2007; Lang et al., 2012) as well as rumen bacterial community composition and function (Pitta et al., 2016) between primiparous and multiparous cows. Most of the milk phenotype-associated OTUs were taxonomically assigned to the phyla Bacteroidetes and Firmicutes. Bacteroidetes was reported to contribute to a majority of metabolic functions in primiparous cows while contributions of Firmicutes and Proteobacteria were more pronounced in multiparous cows (Pitta et al., 2016). This information can be supportive of our observation that B/F ratio in primiparous cows showed a decreased trend over the lactation period. Perhaps an increased proportion of Firmicutes as the primiparous cows advanced through the lactation is required to maximize the contributions of the rumen microbiota to milk phenotype. Difference in the lactation stage of the involved cows in our case is indeed a critical factor for determining stability of the rumen bacterial community. Moreover, the absence of milk phenotype-associated OTUs in primiparous cows is in accordance with previous findings, where more complex interactions, both within and between the microbial domains (as represented by anaerobic fungi, methanogens and bacteria), were observed in multiparous cows than in primiparous cows (Kumar et al., 2015). Therefore, future efforts are required to investigate the stability of the entire rumen microbiome of dairy cows over the lactation period.

Although methanogenic archaea are the only direct methane producers, we did not observe any significant correlation between relative abundance of the archaeal taxa and methane production. It has been reported that high and low methane-emitting sheep could be differentiated by rumen methanogen mRNA rather than the DNA level (Shi et al., 2014). Thus, methane production may be linked to the activity rather than the relative proportion of specific methanogens, advocating the involvement of transcriptomic-based techniques for future investigations of the rumen archaeal community (Poulsen et al., 2013).

### Interaction Network Between Bacterial and Archaeal Taxa

Co-occurrence analysis revealed distinctive associative patterns between bacterial (*Fibrobacter*, *Ruminococcus*, and Lachnospiraceae) and archaeal taxa (unclassified Methanomassiliicoccaceae and *vadinCA11*), indicating their inter-dependence in the rumen microbial ecosystem. *Fibrobacter succinogenes* is important in rumen fiber digestion (Kobayashi et al., 2008), and this fundamental step may provide substrates to other bacteria that are capable of producing H_2_ or methyl-containing compound. Other well-characterized bacterial members, such as *Ruminococcus albus 7* (Christopherson et al., 2014) and *Lachnospira multiparus* (Rode et al., 1981), have the abilities to produce methyl-containing compounds and/or H_2_ from fibrous substrates, thus favoring the growth of methylotrophic methanogens, as represented by members of the Methanomassiliicoccales order. Furthermore, network analysis revealed *Succiniclasticum* as a keystone member of the rumen microbiome. A known isolate from this group, *Succiniclasticum ruminis* (Van Gylswyk, 1995), is capable of converting succinate to propionate as the sole energy-yielding strategy. This step is of critical importance in the rumen fermentation network, and the resulting product propionate is an important precursor for gluconeogenesis in ruminants. Similarly, our observation that S*accharibacteria* had the highest centrality score may be related to its positive correlations with total SCFAs, acetate, and propionate concentrations.

In conclusion, the results from the present study demonstrated the rumen bacterial and archaeal communities of dairy cows to be largely unstable over the lactation period, and unstable OTUs were most often significantly associated with host phenotypic variation. However, we identified 176 stable bacterial OTUs over the lactation period and these were mostly unrelated to host phenotypes, but may possess important conserved ecological and/or metabolic functions. Associations between milk phenotypes and OTUs were detected in the multiparous cows but absent in the primiparous cows as well as higher parity cows had reduced diversity, suggesting that increased milk production in higher parity cows could be associated with specialization of the rumen microbiome. Further research into the stable and unstable rumen microbiome features is needed to characterize associations between host phenotypic variation and changes in the rumen microbiota. Technically, transcriptomic-based approaches should certainly be included to elucidate functional stability or dynamics of the rumen microbiota, e.g., over the lactation period.

## Data Availability Statement

The datasets presented in this study can be found in online repositories. The name of the repository and accession number can be found below: European Molecular Biology Laboratory’s European Bioinformatics Institute (EMBL-EBI) European Nucleotide Archive (ENA), https://www.ebi.ac.uk/ena/browser/view/PRJEB28065.

## Ethics Statement

The animal study was reviewed and approved by The Animal Experiments Inspectorate, Danish Veterinary and Food Administration, Ministry of Environment and Food of Denmark (Approval number 2016-15-0201-00959).

## Author Contributions

PL, OH, and ZZ: conceptualization. PL, OH, and SN: supervision. JL and ZZ: rumen sample collection. PL, JL, and GD: host phenotype data creation. ZZ, GD, and SN: rumen microbiome analysis. ZZ: writing – original draft. All authors contributed to the article and approved the submitted version.

## Conflict of Interest

The authors declare that the research was conducted in the absence of any commercial or financial relationships that could be construed as a potential conflict of interest.

## Footnotes

1 https://greengenes.secondgenome.com

2 https://www.ebi.ac.uk/ena/data/view/PRJEB28065

## Supplementary Material

Supplementary Figure 1 Venn diagram showing OTU distribution. Shared OTUs between milk phenotype-associated (MPA) OTUs (38 OTUs), stable OTUs (176 OTUs), and a total number of 6363 bacterial OTUs, are illustrated in Venn diagram, indicating that only 2 OTUs, assigned to unclassified Firmicutes and unclassified Bacteroidetes, are shared between the MPA and stable OTUs.

Supplementary Figure 2 SCFA proportions over the lactation period. The relative proportions of individual SCFAs (A: Acetate; P: Propionate; B: Butyrate) were plotted against days in milking ranging from day 13 to day 320. Major SCFA components are designated as open circles with distinct colors. Repeatability of individual SCFA proportions (acetate, propionate and butyrate) are 0.19, 0.11, and 0.21, respectively.
