# Supplementary figures and images for: Stability Assessment of the Rumen Bacterial and Archaeal Communities in Dairy Cows Within a Single Lactation and Its Association With Host Phenotype
Source: Front Microbiol. 2021 Apr 6;12:636223. doi: 10.3389/fmicb.2021.636223 (PMC8076905; doi:10.3389/fmicb.2021.636223)

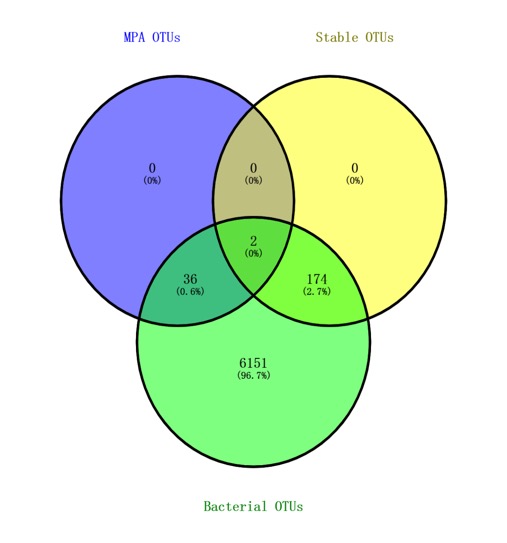

Supplement: Supplementary Figure 1 — Venn diagram showing OTU distribution. Shared OTUs between milk phenotype-associated (MPA) OTUs (38 OTUs), stable OTUs (176 OTUs), and a total number of 6363 bacterial OTUs, are illustrated in Venn diagram, indicating that only 2 OTUs, assigned to unclassified Firmicutes and unclassified Bacteroidetes, are shared between the MPA and stable OTUs. [file Image_1.jpg]

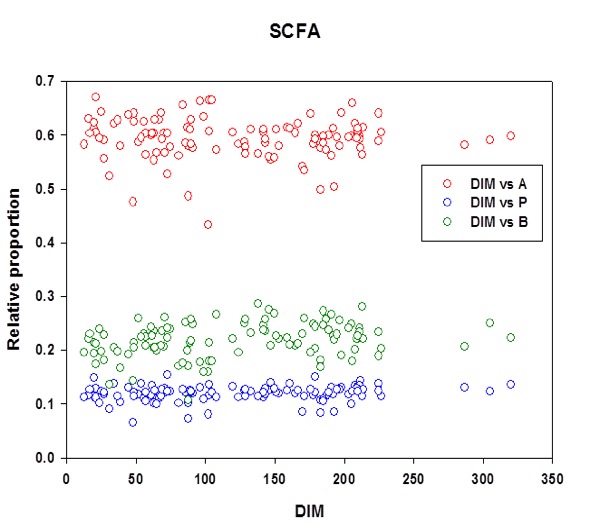

Supplement: Supplementary Figure 2 — SCFA proportions over the lactation period. The relative proportions of individual SCFAs (A: Acetate; P: Propionate; B: Butyrate) were plotted against days in milking ranging from day 13 to day 320. Major SCFA components are designated as open circles with distinct colors. Repeatability of individual SCFA proportions (acetate, propionate and butyrate) are 0.19, 0.11, and 0.21, respectively. [file Image_2.jpg]
